# Profound Change in Soil Microbial Assembly Process and Co-occurrence Pattern in Co-inoculation of *Bradyrhizobium japonicum* 5038 and *Bacillus aryabhattai* MB35-5 on Soybean

**DOI:** 10.3389/fmicb.2022.846359

**Published:** 2022-03-18

**Authors:** Yubin Zhao, Dawei Guan, Xu Liu, Gui-Feng Gao, Fangang Meng, Bingqiang Liu, Pengfei Xing, Xin Jiang, Mingchao Ma, Fengming Cao, Li Li, Jun Li

**Affiliations:** ^1^Institute of Agricultural Resources and Regional Planning, Chinese Academy of Agricultural Sciences, Beijing, China; ^2^State Key Laboratory of Soil and Sustainable Agriculture, Institute of Soil Science, Chinese Academy of Sciences, Nanjing, China; ^3^Soybean Research Institute, Jilin Academy of Agricultural Sciences, Jilin, China; ^4^Institute of Cereal and Oil Crops, Hebei Academy of Agricultural and Forestry Sciences, Hebei, China

**Keywords:** *Rhizobium*, *Bacillus*, co-inoculation, microbial assembly process, co-occurrence pattern

## Abstract

Rhizosphere microbial communities are vital for plant growth and soil sustainability; however, the composition of rhizobacterial communities, especially the assembly process and co-occurrence pattern among microbiota after the inoculation of some beneficial bacteria, remains considerably unclear. In this study, we investigated the structure of rhizomicrobial communities, their assembly process, and interactions contrasting when *Bradyrhizobium japonicum* 5038 and *Bacillus aryabhattai* MB35-5 are co-inoculated or *Bradyrhizobium japonicum* 5038 mono-inoculated in black and cinnamon soils of soybean fields. The obtained results indicated that the Chao and Shannon indices were all higher in cinnamon soil than that in black soil. In black soil, the co-inoculation increased the Shannon indices of bacteria comparing with that of the mono-inoculation. In cinnamon soil, the co-inoculation decreased the Chao indices of fungi comparing with that of mono-inoculation. Compared with the mono-inoculation, the interactions of microorganisms of co-inoculation in the co-occurrence pattern increased in complexity, and the nodes and edges of co-inoculation increased by 10.94, 40.18 and 4.82, 16.91% for bacteria and fungi, respectively. The co-inoculation of *Bradyrhizobium japonicum* 5038 *and Bacillus aryabhattai* MB35-5 increased the contribution of stochastic processes comparing with *Bradyrhizobium japonicum* 5038 inoculation in the assembly process of soil microorganisms, and owing to the limitation of species diffusion might restrict the direction of pathogenic microorganism movement. These findings support the feasibility of rebuilding the rhizosphere microbial system via specific microbial strain inoculation and provide evidence that the co-inoculation of *Bradyrhizobium japonicum* 5038 and *Bacillus aryabhattai* MB35-5 can be adopted as an excellent compound rhizobia agent resource for the sustainable development of agriculture.

## Introduction

Rhizobia have been identified as symbiotic nitrogen-fixing bacteria that fix atmospheric nitrogen to the soil, thus forming an important nitrogen input for the sustainability of the agricultural ecosystem (Stewart, 1967; Kumar et al., 2019); in addition, rhizobial inoculants can be the biocontrol for a number plant diseases and minimize the prevalence of some pathogenic bacteria (Nimnoi et al., 2011; Neda, 2021), which is an effective approach to reducing the application of chemical fertilizers and pesticides. The close association of *Rhizobium* and soybean provide sufficient mutual benefits. However, owing to the specific symbiotic matching characteristics between different rhizobia and plants host (Wang et al., 2009), environmental stress (Thilakarathna and Raizada, 2017), and the competition of native rhizobial strains (Schumpp and Deakin, 2010), the multivariate mechanism of *Rhizobium* inoculation on nodulation of soybean is unclear. Several studies indicated that the co-inoculation of some functional plant growth-promoting rhizobacteria (PGPR) with rhizobia could increase the colonization ability of rhizobia and promote the nodulation and nitrogen fixation of soybean (Srinivasan et al., 1997; Moretti et al., 2020; Roper et al., 2020). Concurrently, various microbial inoculants can dissolve phosphorus and potassium, increase the content of soluble phosphorus, potassium, and other mineral elements in the soil, and further increase soybean yield (Jun et al., 2019; Iturralde et al., 2020; Ju et al., 2020). Although the positive effects of PGPR on plant growth have been well known for decades, the underlying molecular mechanisms of plant growth promotion, especially in soil microbial communities, remain unexplored.

To date, *Bacillus* has been the most extensively studied PGPR. *Bacillus amyloliquefaciens* or *B. subtilis* added in wheat (Mahmud et al., 2021), cucumber (Bi et al., 2019; Wang et al., 2021), tomato (Qin et al., 2017; Wan et al., 2018), and Populus alba L. (Chen et al., 2020) all exhibited significant impact on bacterial diversity and its composition. These reports verify several effects of growth promotion in microbial communities via the inoculation of common and well-researched Bacillus, such as *B. amyloliquefaciens* and *B. subtilis*. However, the structure of microbial communities when exogenous *B. aryabhattai* and the underlying mechanisms are introduced in agricultural soil remain unclear. *B. aryabhattai strain* B8W22 was initially isolated from cryotubes used to collect air samples from the Earth’s stratosphere at an altitude between 27 and 41 km (Shivaji et al., 2009), and strains of this species was later isolated from rhizosphere soil in several parts of the world (Lee et al., 2012; Pailan et al., 2015; Yan et al., 2016). Some studies indicated that *B. aryabhattai* can be a valuable organism for its incorporation into biofertilizers and other soil amendments to improve crop productivity and tolerance toward pesticides by regulating the homeostasis of several phytohormones and producing different gibberellic acids (Park et al., 2017). In addition, the effects of the changes in *B. aryabhattai* on soil microorganisms have also been studied. *B. aryabhattai* broth has been supplemented with insoluble zinc compounds. In another study, further inoculation substantially decreased the rhizosphere soil pH and increased dehydrogenase, β-glucosidase, auxin production, microbial respiration, and microbial biomass in the rhizosphere soils of soybean and wheat crops (Ramesh et al., 2014). When *B. aryabhattai* was added in Chinese cabbages, it changed soil fungal community structure considerably, and the control efficiency of clubroot was 76.92% (Liu et al., 2018). The inoculation of *B. aryabhattai* could alter the soil microbial community; however, deeper microbial mechanisms when *Rhizobium* and *B. aryabhattai* co-inoculate and interact lack in-depth research.

The assembly process and co-occurrence pattern are two advanced and prevalent approaches currently adopted in ecology for exploring microbial mechanisms, and they have obtained extensive research results (Zhou et al., 2017a; Tripathi et al., 2018; Chen et al., 2019). It is generally accepted that deterministic and stochastic processes were simultaneously presented for community assembly in various ecosystem types (Feng et al., 2018; Shi et al., 2018; Sengupta et al., 2019; Zhao et al., 2019). Furthermore, the co-occurrence pattern indicates that soil microbial microorganisms interact with each other through the links of nodes and edges (Faust and Raes, 2012). To elucidate the links of community assembly processes and the ecological strategies of co-occurring species, two different but complementary category theories, the niche-based theory and the neutral theory, were raised to perform quantitative analysis and examine the contributions of deterministic and stochastic processes in the microbial community assembly. The niche-based theory is based on the differences in ecological niches of co-occurring species (Hubbell, 2011), and the neutral theory relies on dispersal and stochastic demographic processes (Silvertown, 2004). A recent study demonstrated that in soybean fields, deterministic processes play a dominant role in bacterial community assembly (Zhang et al., 2018). Moreover, in different soybean cultivars, the assembly process and co-occurrence networks of rhizobacterial communities were determined to be different (Zhong et al., 2019). Soil type can also alter the co-occurrence of microbial communities (Liu et al., 2019). Previous studies also indicate that the changes in the assembly process and co-occurrence pattern are related to the downstream impacts on the function of the system (Tedersoo et al., 2014; Maynard et al., 2019). Hence, revealing the details of the complex interactions and assembly process is potentially crucial in developing novel strategies for sustainable agriculture and comprehending the ecological mechanisms of the introduced *Rhizobium* and *Bacillus*.

Considering spatial heterogeneity, the effect of different species of functional PGPR inoculated on soil bacterial and fungal communities was conducted in two soil types for two cultivars in a major soybean production region in China. Soil microbial diversity, co-occurrence pattern, and assembly process of co-inoculation of *Bradyrhizobium japonicum* 5038 *and Bacillus aryabhattai* MB35-5 were collectively investigated to verify the growth-promoting effect of co-inoculating *Bradyrhizobium japonicum* 5038 and *Bacillus aryabhattai* MB35-5 for the first time.

## Materials and Methods

### Experimental Design

To explore the effects of co-inoculating *Bradyrhizobium japonicum* 5038 *and Bacillus aryabhattai* MB35-5 on soybean, no-inoculation (100% chemical fertilizer), one-inoculation (75% chemical fertilizer + Bradyrhizobium japonicum 5038), and two inoculation (75% chemical fertilizer + *Bradyrhizobium japonicum* 5038 + *Bacillus aryabhattai* MB35-5) treatments were conducted in black and cinnamon soils with a total of six samples (B0, B1, B2, C0, C1, and C2) marked in this study. The doses of chemical fertilizers were 100 kg N/ha, 100 kg P_2_O_5_/ha, and 100 kg K_2_O/ha for no-inoculation treatment, and 75 kg N/ha, 75 kg P_2_O_5_/ha, and 75 kg K_2_O/ha for one-inoculation and two inoculation treatments. The chemical fertilizer used in this study was composed of urea (CON_2_H_4_), Ca(H_2_PO_4_)_2_⋅H_2_O and K_2_SO_4_.

### Description of the Applied Bacterial Strains

The *Bradyrhizobium japonicum* 5038 (CGMCC16747) and *Bacillus aryabhattai* MB35-5 used in this study were screened in our laboratory. *Bradyrhizobium japonicum* 5038, a nitrogen-fixing strain that can tolerate water stress, was screened from 160 purified strains of *Bradyrhizobium* that had previously been isolated from the root nodules of different Asiatic soybean cultivars. This research was published by Zhu et al. (2022). *Bacillus aryabhattai* MB35-5 was screened from 204 purified strains of bacteria, which had previously been isolated from soils in different rice and wheat fields, that were mostly located in 16 Chinese cities. This strain is resistant to salt, alkali, acid and anaerobic conditions. It increased the root dry matter of corn and maize by 24.02 and 16.21%, respectively, compared with the control *Silicate bacteria* 3016. These results have already been used to obtain a Chinese invention patent (CN112772678B).

### Seed Preparation

Single colonies of *Bradyrhizobium japonicum* 5038 were cultured in 100 ml liquid yeast extract mannitol broth (YMB) containing 10.0 g mannitol, 0.2 g MgSO4⋅7H_2_O, 0.1 g NaCl, 0.5 g K_2_HPO_4_, 0.5 g yeast extract, 1000 ml H_2_O (pH = 7.0). Single colonies of *B. aryabhattai MB35-5* were cultured in 100 ml liquid R2A containing 0.5 g yeast extract, 0.5 g tryptone, 0.5 g glucose, 0.3g K_2_HPO_4_, 5.0 g MgO_3_Si, 1000 ml H_2_O (pH = 7.0). The cells were incubated at 30°C with shaking at 180 rpm until reached to the value of OD_600_ for 0.6–0.8. The cells were incubated at 30°C with shaking at 180 rpm until they reached OD600 levels of 0.6–0.8. For the one-inoculation treatment, 3 ml of active culture of *Br. japonicum* 5038 was mixed with 1 kg of soybean seeds. When both strains were co-inoculated simultaneously, 1.5 ml of each activated bacterial liquid was mixed with 1 kg of soybean seeds. All the inoculated seeds were air-dried 2–3 h in the shade before planting.

### Soil Sampling

Black soil samples were collected from a trial site located in Songyuan, Jilin Province (N 45°13′, E 124°82′), whereas cinnamon soil samples were collected from a trial site located in Shijiazhuang, Hebei Province (N 38°05′, E 114°50′). These sites are two experimental stations belonging to China’s agricultural research system. Soybean cultivars were Jiyu47 and Jidou12 for black soil and cinnamon soil, respectively. Soil samples were collected in September 7 and August 22 of 2020 at soybean early pod stage. Rhizosphere soil at a depth of 10–20 cm (plow layer) was collected, gently shaking off the soil which loosely adhered to the roots, retaining the soil tightly adhered to roots, collecting the soil by brush, and mixing to obtain one composite soil sample per plot. Then, we randomly selected five samples from each plot for the different treatments, and a total of 30 samples were collected. After the collection, all soil samples were stored in an icebox and transferred to a laboratory for examination. Each sample was separated into two parts: one part was stored at −80°C for biological analysis and the other part was air-dried in the shade for physical and chemical analyses.

### Soil Properties

The soil nitrate-nitrogen (NN) and ammonium-nitrogen (AN) contents were determined using samples of fresh soil, whereas the other soil properties were analyzed after drying soil samples at 25 ± 5°C and passing them through a 1.0 mm mesh. The soil pH was measured using a pH meter with soil to water ratio of 1:1 (Li et al., 2013). The NN and AN contents were determined via flow injection analysis (Mulvaney, 1996), whereas the soil total nitrogen (TN) and organic carbon (TC) levels were determined according to the procedure presented by Strickland and Sollins (1987). The alkali-hydrolyzable nitrogen (HN) was measured by an alkaline extraction method (alkaline potassium permanganate method). The available phosphorus (AP) was quantified using the NaHCO_3_^–^ extraction method, whereas the available potassium (AK) was quantified using the NH_4_OAc extraction method and a flame photometer (FP640, Shanghai, China) (Mulvaney, 1996). In addition, the soil metallic elements (Na, Ca, and Mg) were measured according to Acero et al. (2006).

### Properties of Soybean Nodules

When the soil sample was collected, at the same time, five completely plant roots of each treatment was collected and transported to the lab. Then, we selected the relatively full and suitable size nodules for counting and then weighted the dry weight of the nodules at 80°C until we reached a constant weight. The increasing numbers and dry matter of nodules that enable the inoculation of different PGPR species are illustrated in Supplementary Figure S1.

### DNA Extraction and qPCR

We extracted total DNA from rhizosphere soil with a Power Soil DNA Isolation Kit (MOBIO Laboratories Inc., Carlsbad, CA, United States) according to the manufacturer’s instructions. The DNA quality and concentration (A260/A280) were estimated using a NanoDrop ND-1000 UV-Vis Spectrophotometer (Thermo Scientific, Rockwood, TN, United States). Each DNA sample was stored at −80°C until further analysis. Subsequently, bacteria were analyzed by sequencing the V3-V4 hypervariable region of the 16S ribosomal RNA (rRNA) gene. The V3-V4 region was amplified using universal primers, 338F and 806R (338F: 5′-ACTCCTACGGGAGGCAGCA-3′ and 806R: 5′-GGACTACHVGGGTWTCTAAT-3′) (Xu et al., 2016). The PCR mixtures contain 5 × TransStart FastPfu buffer 4 μL, 2.5 mM dNTPs 2 μL, forward primer (5 μM) 0.8 μL, reverse primer (5 μM) 0.8 μL, TransStart FastPfu DNA Polymerase 0.4 μL, BSA 0.2 μL, template DNA 10 ng, and finally ddH_2_O up to 20 μL. The hypervariable regions of the fungal 18S rRNA gene were amplified with the 817F and 1196R primers (817F: 5′-TTAGCATGGAATAATRRAATAGGA-3′ and 1196R: 5′-TCTGGACCTGGTGAGTTTCC-3′) using a thermocycler polymerase chain reaction (PCR) system (GeneAmp 9700, ABI, California, United States) (Rousk et al., 2010). The PCR mixtures contain 10 × buffer 2 μL, 2.5 mM dNTPs 2 μL, forward primer (5 μM) 0.8 μL, reverse primer (5 μM) 0.8 μL, rTaq Polymerase 0.2 μL, BSA 0.2 μL, template DNA 10 ng, and finally ddH_2_O up to 20 μL. The PCR amplification of 16S rRNA gene was performed as follows: initial denaturation at 95°C for 3 min, followed by 30 cycles of denaturing at 95°C for 30 s, annealing at 55°C for 30 s and extension at 72°C for 45 s, and single extension at 72°C for 10 min, and end at 4°C.

### Sequencing Data Processing

The PCR products were utilized to purify and generate the amplicon libraries; subsequently, the obtained data were analyzed using QIIME pipeline version 1.8.0. We excluded low-quality reads in an initial quality-filtering step. Chimeric sequences were then identified using UCHIME. Operational taxonomic units (OTUs) were defined via clustering at 97% similarity. The rarefaction and diversity indices were calculated after clustering the OTUs. The taxonomy of each 16S rRNA gene sequence was analyzed by the ribosomal database project classifier^1^ against the silva138/16s_bacteria 16S rRNA database and Unite8.0/its_fungi as references with a confidence threshold of 70% (Amato et al., 2013). Data were analyzed using a free online platform, Majorbio I-Sanger Cloud Platform^2^. The raw data were deposited in the short reads archive database of the National Center for Biotechnology Information (SRA accession: PRJNA788980, PRJNA788360).

### Statistical Analysis

The network analysis was designed based on all the 30 samples at the phylum and class levels of bacteria and fungi. To explore all the pairwise associations, correlation scores (Spearman’s correlation), OTUs with relative abundances less than 0.005% or in less than five soil samples were discarded. The relationship among OTUs was examined by Pearson’s correlation, Spearman’s correlation, Bray–Curtis dissimilarity, and Kullback–Leibler dissimilarity using R (version 3.6.3). The *P*-values of the five methods were integrated using the Brown method, and the correlations | *P*| > 0.7 were retained for the downstream procedure. The resulting correlations were imported into the Gephi platform and then visualized by the Fruchterman–Reingold algorithms.

To determine the potential importance of stochastic processes on community assembly, we adopted a neutral community model (NCM) to predict the relationship between OTU detection frequencies and their relative abundance across the wider metacommunity, which were performed using R (version 3.6.3) and the program presented by Chen et al. (2019). The assembly processes of bacterial and fungal communities were evaluated by calculating the nearest taxon index and beta nearest taxon index (betaNTI) using the “ses.mntd” function in a picante package (Stegen et al., 2012; Xun et al., 2019). When | betaNTI| < 2, the contribution was considered a stochastic process, and when | betaNTI| > 2 the shifts in community composition were deterministic processes.

Soil physicochemical property data were analyzed using Microsoft Excel 2010 pro and SPSS version 20. A Mantel test was adopted to determine the significance of the relationship between soil physicochemical properties and community structure using QIIME. A random forest model was used to identify the bacterial and fungal taxa on the order level that accurately predict the co-inoculation of *Bradyrhizobium japonicum* 5038 and *Bacillus aryabhattai* MB35-5 by adopting the random forest package (importance = TRUE, proximity = TRUE, ntree = 1000). We further adopted the variation partitioning analysis (VPA) with adjusted *R*^2^ coefficients based on the redundancy analysis (RDA) and partial Mantel test to quantify the relative effects of environmental and spatial factors in shaping community composition (R version 3.3.1).

## Results

### Effect of Co-inoculating *Bradyrhizobium japonicum* 5038 and *Bacillus aryabhattai* MB35-5 on Bacterial and Fungal Communities

**FIGURE 1:**
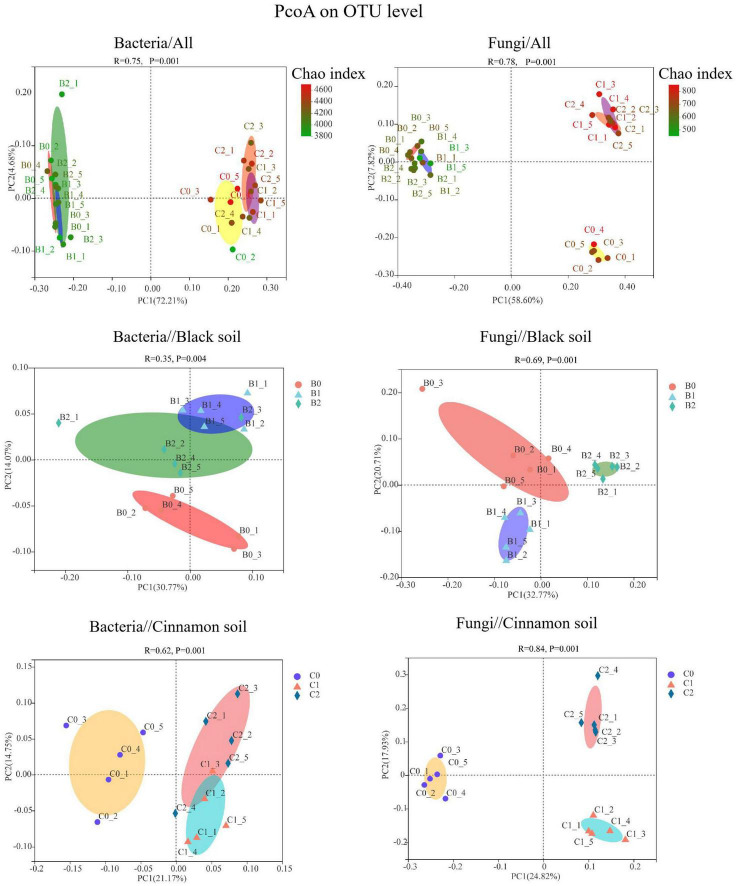
PCoA based on Bray–Curtis dissimilarities showing the difference in bacterial and fungal community in black soil and cinnamon soil. B0, B1, and B2 means no-inoculation, inoculation of *Bradyrhizobium japonicum* 5038, and co-inoculation of *Bradyrhizobium japonicum* 5038 and *Bacillus aryabhattai* MB35-5 in the black soil, and C0, C1, and C2 means no-inoculation, inoculation of *Bradyrhizobium japonicum* 5038, and co-inoculation of *Bradyrhizobium japonicum* 5038 and *Bacillus aryabhattai* MB35-5 in the cinnamon soils.

An analysis of similarities indicated the inoculation treatments that produced substantial effects on the diversity of bacterial (*R*^2^ = 0.76, *p* < 0.01) and fungal (*R*^2^ = 0.78, *p* < 0.01) communities relative to the OTU level (Figure 1). The Chao and Shannon indices of the three treatments were all higher in cinnamon soil than that in black soil, respectively. In black soil, the co-inoculation of *Bradyrhizobium japonicum* 5038 and *Bacillus aryabhattai* MB35-5 increased the Shannon indices of bacteria comparing with that of the *Bradyrhizobium japonicum* 5038 mono-inoculated treatment. In cinnamon soil, the co-inoculation of *Bradyrhizobium japonicum* 5038 and *Bacillus aryabhattai* MB35-5 decreased the Chao indices of fungi comparing with that in *Bradyrhizobium japonicum* 5038 mono-inoculated treatment (Supplementary Figure S3).

**FIGURE 2:**
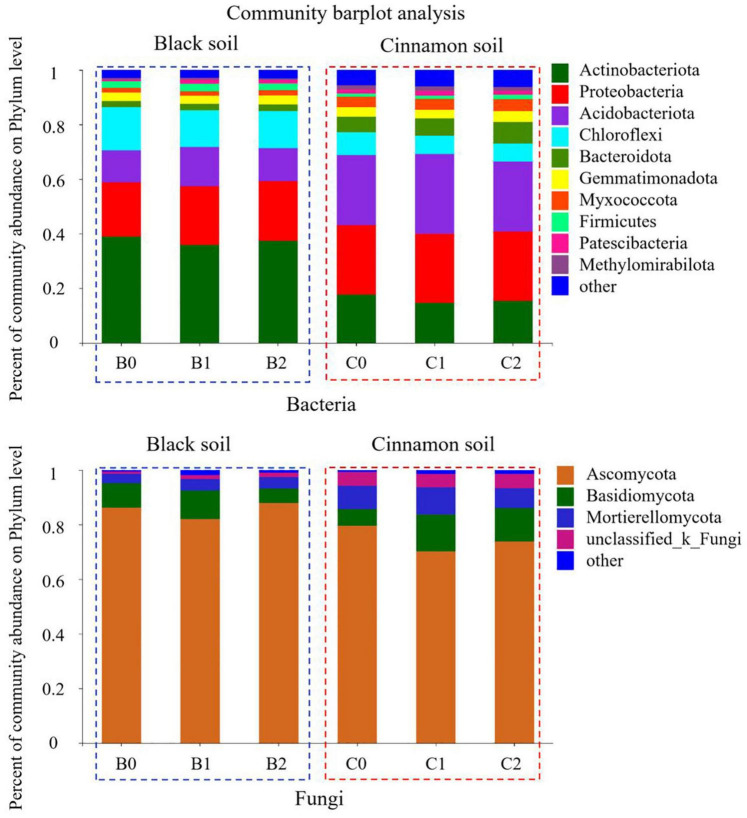
The relative abundance of top 10 phyla for each three treatments in black and cinnamon soil.

A total of 7592 rarefied OTUs from 40 phyla were identified for bacteria. The top 10 most abundant phyla were *Actinobacteria* (37.37, 15.84%), *Proteobacteria* (21.03, 25.30%), *Acidobacteria* (12.68, 26.82%), *Chloroflexi* (14.31, 7.14%), *Bacteroidetes* (2.34, 6.71%), *Gemmatimonadetes* (3.15, 3.53%), *Myxococcota* (1.77, 4.07%), *Firmicutes* (2.61, 1.52%), *Patescibacteria* (1.13, 1.59%), and *Methylomirabilota* (0.59, 1.49%) in the black and cinnamon soils, respectively (Figure 2). To identify the most important predictors for shaping bacterial communities, 13 soil physicochemical properties were measured (Supplementary Table S1). The Mantel tests demonstrated that the bacterial communities were significantly (*p* < 0.05) correlated with TC, HN, and Ca for black soil and TC, TN, NN, AN, Ca, and pH for the cinnamon soil (Supplementary Table S2). Among these variables, HN (Mantel *r* = 0.36, *p* < 0.05) and NN (Mantel *r* = 0.58, *p* < 0.05) exhibited the strongest correlation with the bacterial community structure for black and cinnamon soil, respectively. Principal coordinates analysis (PCoA) results indicated that the bacterial communities were divided along the soil type and number of bacteria inoculation in axes 1 and 2, which could explain 72.21 and 4.68% of the total variations, respectively (Figure 1). The relative abundance of most of the top 10 phyla was significantly (*p* < 0.05) correlated with the main soil physicochemical properties. Among the soil physicochemical properties, pH and AK exhibit the significant correlation (*p* < 0.05) with more bacterial communities in black soil (Supplementary Table S4); TN and Mg exhibit the significant correlation (*p* < 0.05) with more bacterial communities in the cinnamon soil (Supplementary Table S5). Random forest analysis was adopted to identify biomarkers at the family level that are strongly associated with the PGPR species (Supplementary Figure S4). We determined that several of them were enriched by the increase of *Bradyrhizobium japonicum* 5038 and *Bacillus aryabhattai* MB35-5, including *Pezizaceae*, *Aspergillaceae*, *Ascodesmidaceae*, and *Rhizophlyctidaceae*.

A total of 2405 rarefied OTUs from 16 phyla were identified for fungi. The top three most abundant phyla were *Ascomycot*a (85.38, 74.54%), *Basidiomycota* (8.23, 10.54%), and *Mortierellomycota* (3.97, 8.63%) in the black and cinnamon soils, respectively (Figure 2). Moreover, the same phenomenon emerged in the two soil types, such that with the increase in the species of inoculated bacteria, *Ascomycota* first decreased and then increased, whereas *Basidiomycota* increased first before decreasing. In other words, the inoculation with *Bradyrhizobium japonicum* 5038 reduced *Ascomycota* and increased *Basidiomycota*, and then the addition of *Bacillus* increased *Ascomycota* and decreased *Basidiomycota*. Mantel tests demonstrated that the fungal communities were significantly (*p* < 0.05) correlated with TC, NN, AN, AP, TK, Na, Ga, and Mg for the black soil and with TC, TN, NN, AP, AK, Na, Ca, and pH for the cinnamon soil (Supplementary Table S2). Among these variables, NN (Mantel *r* = 0.48, *p* < 0.05; *r* = 0.58, *p* < 0.05) exhibited the strongest correlation with the fungal community structure for both black and cinnamon soils. PCoA results showed that the fungal communities were divided along the soil type and number of inoculation of bacteria in axes 1 and 2, which could explain 58.60 and 7.82% of the total variations, respectively (Figure 1). The relative abundance of most of the top three phyla was significantly (*p* < 0.05) correlated with the main soil physicochemical properties. Among the soil physicochemical properties, HN and TN are more significantly correlated with the microbial communities in the black soil (Supplementary Table S4); pH and Na have a more significant correlation with the microbial communities in the cinnamon soil (Supplementary Table S5). *Rhizophlyctidales*, *Auriculariales*, *Diversisporales*, *Capnodiales*, and other fungi in the order level were analyzed as biomarkers that were strongly associated with the co-inoculation of *Bradyrhizobium japonicum* 5038 and *Bacillus aryabhattai* MB35-5 (Supplementary Figure S4).

### Effect of Co-inoculating *Bradyrhizobium japonicum* 5038 and *Bacillus aryabhattai* MB35-5 on the Co-occurrence Pattern of the Bacterial and Fungal Communities

**FIGURE 3:**
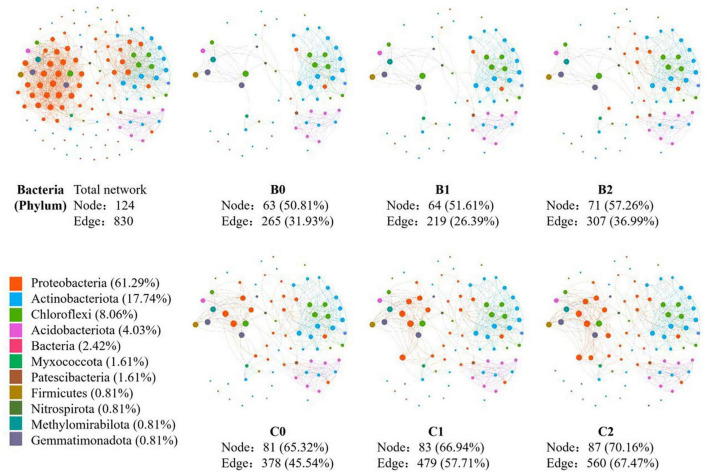
The co-occurrence pattern of total bacterial community and each sub-network fallowing co-inoculation of *Bradyrhizobium japonicum* 5038 and *Bacillus aryabhattai* MB35-5 in black and cinnamon soil.

**FIGURE 4:**
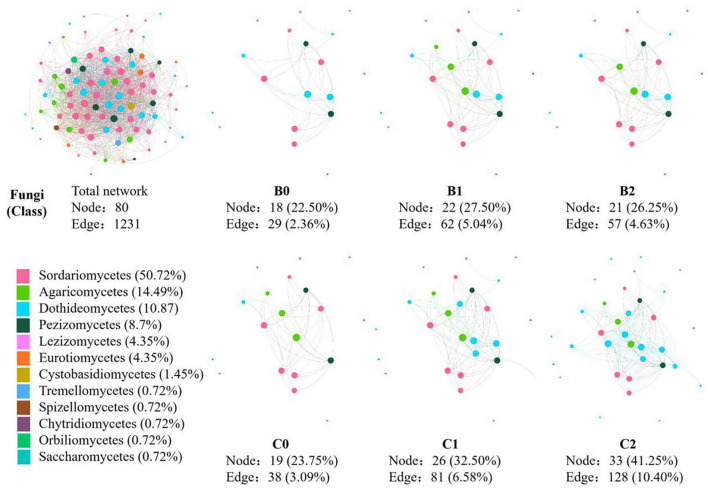
The co-occurrence pattern of total fungal community and each sub-network fallowing co-inoculation of *Bradyrhizobium japonicum* 5038 and *Bacillus aryabhattai* MB35-5 in black and cinnamon soil.

In the co-occurrence network constructed by all 30 soil samples, 124 nodes and 1231 edges were identified for soil bacteria, whereas 80 nodes and 830 edges were identified for soil fungi (Figures 3, 4). In total, 12 sub-network analyses were also performed, as illustrated in Figures 3, 4 for bacteria and fungi, respectively, which showed that as the *Bradyrhizobium japonicum* 5038 and *Bacillus aryabhattai* MB35-5 inoculated, the nodes and edges increase. In other words, the inoculation of *Bradyrhizobium japonicum* 5038 and *Bacillus aryabhattai* MB35-5 could increase the complexity of the co-occurrence pattern for both bacteria and fungi. The rich microorganisms in the sub-network were different in the two soil types, and the complexity of the co-occurrence pattern in the cinnamon soil was higher than that in the black soil, which is especially reflected in the increase in the amount of *Proteobacteria* and *Dothideomycetes*.

In total, 21 and 3 key species of bacteria and fungi within the network were identified (Supplementary Figure S5), which included *Gymnoascus*, *Cyphellophora*, *Azospirillales*, and several other microorganisms (Supplementary Table S6). Moreover, we identified several key species as biomarkers, *Cyphellophora* (*R*^2^ = 0.484, *P* = 0.002) in the black soil and *Solirubrobacteraceae* (*R*^2^ = 0.379, *P* = 0.009) in the cinnamon soil, which was exhibited a strong negative association with PGPR numbers, and *Leucoagaricus* (*R*^2^ = 0.425, *P* = 0.005) in the cinnamon soil, which exhibited a strong positive correlation with PGPR species (Supplementary Figures S6, S7).

The relationship between network characteristics and the physical and chemical properties of soil is illustrated in Supplementary Figure S8. HN, TN, and TK were significantly positive with the number of nodes in 12 sub-networks, whereas the Ca and Mg contents were also important factors with a significant negative relationship with the number of bacteria and fungi nodes, respectively. In addition, environmental factors via the degree of bacteria network and average path length of the fungi network affected the co-occurrence pattern.

### Community Assembly Processes in Response to the Co-inoculation of *Bradyrhizobium japonicum* 5038 and *Bacillus aryabhattai* MB35-5

**FIGURE 5:**
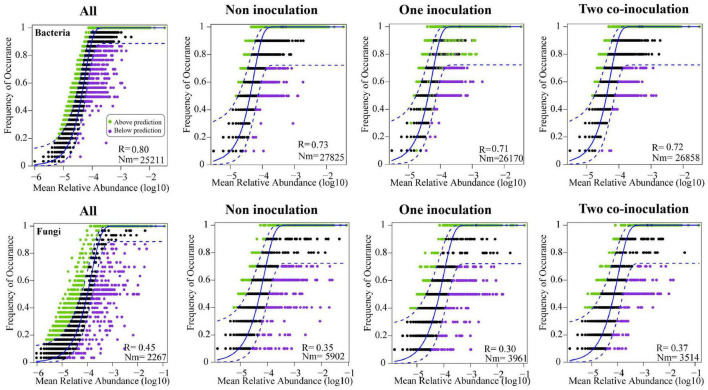
Fit of the neutral community model (NCM) of community assembly. The predicted occurrence frequencies for non-inoculation, one inoculation, two co-inoculation, and all representing soil bacterial and fungal communities. The solid blue lines indicate the best fit to the NCM, and the dashed blue lines represent 95% confidence intervals around the model prediction. OTUs that occur more or less frequently than predicted by the NCM are shown in different colors. Nm indicates the metacommunity size times immigration, R indicates the fit to this model.

**FIGURE 6:**
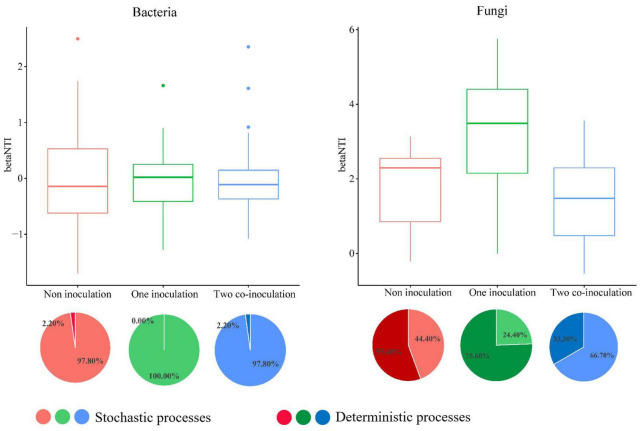
The community assembly processes by fitting niche-based theories. The relationship between inoculation of *Bradyrhizobium japonicum* 5038 *and Bacillus aryabhattai* MB35-5 and betaNTI. The top 50 and 100 abundance OTU were chose for constructing phylogenetic tree. Pie plots showing the relative contribution of each ecological process in community assembly response along the inoculation of *Bradyrhizobium japonicum* 5038 and *Bacillus aryabhattai* MB35-5.unity model of community assembly.

The NCM successfully estimated a large fraction of the relationship between the occurrence frequency of OTUs and their relative abundance variations (Figure 5), with 73, 71, and 72% of explained bacterial community variance for non-inoculation, one-inoculation, and two co-inoculation treatments, respectively. The obtained results indicated that stochastic processes were crucial in shaping the bacterial community assembly, and the co-inoculation of *Bradyrhizobium japonicum* 5038 and *Bacillus aryabhattai* MB35-5 can increase the stochastic processes for driving the microbial community assembly. The contribution of the stochastic and deterministic processes with the inoculation of the *Bradyrhizobium japonicum* 5038 and *Bacillus aryabhattai* MB35-5 was also in agreement with these results (Figure 6). Although the relationships between the occurrence frequency of OTUs and their relative abundance variations were 35, 30, and 37%, we acknowledge that the co-inoculation of *Bradyrhizobium japonicum* 5038 and *Bacillus aryabhattai* MB35-5increased the stochastic processes for the fungi community assembly. The contribution of the deterministic processes in fungi was higher than those in bacteria, which also indicates that the deterministic process plays a more significant role in driving the fungal community assembly (Figure 6). The Nm-value was lower for bacterial taxa with the one-inoculation treatment of *Bradyrhizobium japonicum* 5038 (Nm = 26 170) and two co-inoculation treatment of *Bradyrhizobium japonicum* 5038 and *Bacillus aryabhattai* MB35-5 (Nm = 26 858) than the non-inoculation treatment (Nm = 27 825). This phenomenon was more significant for the fungi community, and the Nm-value decreased to the one-inoculation treatment of *Bradyrhizobium japonicum* 5038 (Nm = 3961) and two co-inoculation treatments of *Bradyrhizobium japonicum* 5038 and *Bacillus aryabhattai* MB35-5 (Nm = 3514) from non-inoculation (Nm = 5902). These results indicate that the species diffusion decreased after inoculating PGPR.

## Discussion

### Co-inoculation of *Bradyrhizobium japonicum* 5038 and *Bacillus aryabhattai* MB35-5 Increased the Complexity of the Co-occurrence Pattern

Recently, the co-occurrence pattern in ecology has been widely adopted to explore the ecological interactions among microbial communities across various ecosystems (Williams et al., 2014; Fan et al., 2021; Gao et al., 2021). In this study, the obtained results indicated that the network of the microbial community in two soil types after inoculation of *Bradyrhizobium japonicum* 5038 became complex, and some functional microorganisms increased, e.g., the nitrogen-fixing *Azospirillales* (Supplementary Table S6). Several previous studies also presented similar results, which indicated that in addition to simulating the proliferation of potential beneficial microbes, *Bradyrhizobium japonicum* 5038 inoculation also increased connections in rhizobacterial networks and altered the hub taxa (Zhong et al., 2019; Shang et al., 2021). In another study, the results indicated that compared to grass treatments, legumes increased soil bacterial diversity and the abundance of nitrogen-fixing *Nitrospirae* groups (Zhou et al., 2017b). For fungi, *Bradyrhizobium japonicum* 5038 inoculation in the soybean also facilitated an increase in the number of connections between fungi, including the hub fungi shift to *Phaeosphaeria*, *Sporobolomyces*, *Septoria*, *Edenia*, and *Leptospora* (Xu et al., 2020). Moreover, by building mutants with a defective Nod factor, previous research confirmed that the symbiosis compromised NoeI mutant-inoculation could reduce microbial diversity and co-occurrence interactions, and decrease the abundance of beneficial microbes (Liu et al., 2020). This could be because the inoculated *Bradyrhizobium japonicum* 5038 forms a more symbiotic system with the host plant, which was changed by altering plant root exudates such as flavonoid (White et al., 2015), Catechin (Mommer et al., 2016), and some volatile organic compounds to change the soil physical and chemical environment and influence the prevalence of rhizosphere microorganisms (Vishwakarma et al., 2020).

It is widely acknowledged that *Bacillus* produces highly resistant spores that can survive for prolonged periods in the soil, which are very promising as potential inoculants in agriculture (Kumar et al., 2016; Ferreira et al., 2020; Oleńska et al., 2020). In this study, the nodes and edges of the co-occurrence networks increased with the PGPR species, thus indicating that the co-inoculation of *Bradyrhizobium japonicum* 5038 and *Bacillus aryabhattai* MB35-5 leads to networks with more complexity than the single inoculated *Bradyrhizobium japonicum* 5038 (Figures 3, 4). Previous studies indicated with an increase in the diversity of microorganisms, the interaction relationship becomes stronger, which increases plant tolerance to a range of other abiotic stresses such as heavy metals contamination, water stress (flooding and drought), salinity, and cold (Gerhardt et al., 2009; Ju et al., 2020). After introducing *Bacillus*, pathogenic *Fusarium* certainly decreased to 0.89% from 1.24% in the individually inoculated *Bradyrhizobium japonicum* 5038 treatment in our study. Several studies verified that *Bacillus spp*. could produce 1-aminocyclopropane-1-carboxylate (ACC) to inhibit the stress-related hormone production in the plants and increase biotic stress resistance (Saleem et al., 2007; Hayat et al., 2010). Given the important role of environmental factors in driving microbial communities, the increase in the number of PGPR species can specifically provide microbial populations with more interactions owing to niche differentiation. In addition, keystone species are highly connected with other species within the networks, and they potentially exert a considerable influence on the entire microbial community (Gao et al., 2021). The primary reason for such effect is that different co-inoculated PGPR species can secrete different enzyme systems, such as iron transporter IRT1 (Zhang et al., 2009) and cell wall degrading enzyme (Bell et al., 2019), to increase the nutrient utilization rate. Increasing the supply of nutrients will increase the ecological niche of microorganisms, reduce competition, and allow more microorganisms to participate in the biochemical process. From the random forest analysis, our study also verified that *Bradyrhizobium japonicum* 5038 and *Bacillus aryabhattai* MB35-5 are the main microorganisms that alter the community structure.

Soil is a natural medium for cultivating soil microorganisms, and changes in its physical and chemical properties will trigger changes in the structure and interaction of the microbial community. The physical and chemical properties of different soil types will vary significantly, including the microbial communities (Gowda et al., 2021; Zhu et al., 2021). In the black soil, HN and Ca are the main influencing factors of the bacterial community, and NN and Na are the main influencing factors of the fungal community; in the cinnamon soil, the effects of TC, and NN are more significant in determining the microbial community (Supplementary Tables S2, S3). Interestingly, the same phenomenon emerged after co-inoculating *Bradyrhizobium japonicum* 5038 and *Bacillus aryabhattai* MB35-5 in the two soil types. In other words, the complexity of the network increased after inoculating the PGPR, and as the species of PGPR increased, the interaction between microorganisms also increased. Hence, the co-inoculation of *Bradyrhizobium japonicum* 5038 and *Bacillus aryabhattai* MB35-5 has universal and positive effects on the soil microbial interaction. Collectively, the inoculation of beneficial microorganisms plays a crucial role in maintaining soil ecological health and increasing its resistance. Compound rhizobia agent positively influences plant growth and represents a promising sustainable trend to increase plant production.

### Changes in Co-inoculation of *Bradyrhizobium japonicum* 5038 and *Bacillus aryabhattai* MB35-5 Limitted Species Diffusion

In this study, we examined bacterial and fungal community assembly in agroecosystems across two major soybean production areas throughout north China. Elucidating the relationship between community assembly and species coexistence is fundamental and crucial for understanding ecosystem diversity and functioning. Previous reports indicated that microbial co-occurrence associations tended to be higher when communities were primarily driven by the dispersal limitation relative to species sorting (Jiao et al., 2020). Our findings are in agreement with the finding that the co-inoculation of *Bradyrhizobium japonicum* 5038 and *Bacillus aryabhattai* MB35-5 increased the diversity of bacterial and fungal communities and the complexity of the co-occurrence pattern, at the same time, species diffusion in the community assembly process decreased after inoculating the PGPR species.

The deterministic processes refer to local environmental factors and biotic interactions that are responsible for shaping the microbial community, whereas stochastic processes consider birth, death, speciation, and immigration, which play a key role in shaping the microbial community assembly (Sloan et al., 2006; Zhou and Ning, 2017). In our study, the changes in the stochastic and deterministic processes in the bacterial and fungal communities differed within the inoculation of PGPR. The main assembly processes were stochastic processes for bacteria in the two ecological models, whereas the deterministic process kept increasing for fungi. These results could be attributed to the diversity of the microbial community. Xun et al. (2019) demonstrated that deterministic and stochastic assembly processes are dominant in low- and high-diversity communities, respectively. The bacterial communities exhibit more diversity (Chao index of 4002-4401) than fungi communities (Chao index of 604–814); thus, stochastic processes are dominant for bacteria. Although a previous study demonstrated that deterministic processes play a dominant role in bacterial community assembly in soybean fields (Zhang et al., 2018), the assembly processes of soybean rhizobacterial communities differed in different soybean cultivars and soil types (Liu et al., 2019; Zhong et al., 2019). The inoculation of *Bradyrhizobium japonicum* 5038 and *Bacillus aryabhattai* MB35-5*s* also exerts less impact on the assembly process, because bacteria have more species in sufficient ecological niches. Hence, although the interaction of bacteria increased with the PGPR species (Figure 3), the contribution of the biotic factor was limited. The environmental factor might be the main driving factor for the changes in the bacterial community. In addition, these speculations are well supported by the VPA, which indicated that the environmental factor could explain 86% of the changes in the bacterial community (Supplementary Figure S9).

The contributions of the deterministic assembly process were higher for fungi than bacteria. The VPA demonstrated that the environment factor explains 48% of the changes in the fungal communities, and the 52% residuals, probably owing to the increasing interaction following the co-inoculation of PGPR (Supplementary Figure S9). The inoculation of *Bradyrhizobium japonicum* 5038, due to the increase in the deterministic assembly process (75.60%) (Figure 6), indicated that the environmental factor could exert a more significant effect; thus, the resistance of the fungal community may decrease. When *Bacillus* was added, the contribution of the deterministic assembly processes decreased to 33.30%, whereas the contribution of the stochastic processes increased to 66.70% (Figure 6). *Bacillus* further increased the diversity of fungi, could replenish the ecological niche in time, and competitively prevent the invasion of pathogenic microorganisms (Ongena and Jacques, 2008; Cawoy et al., 2011). Furthermore, the added *Bacillus* further limited the species diffusion and restricted the direction of movement of pathogenic microorganisms. The shift of balance between the deterministic and stochastic processes was also due to different ecological responses of special taxa (e.g., habitat specialists) to the environmental changes under a specific situation (Leibold et al., 2017; Gad et al., 2020). For example, the abundance of *Cyphellophora* with a significant relationship with the PGPR species decreased after the inoculation of *Bradyrhizobium japonicum* 5038 and *Bacillus aryabhattai* MB35-5 in black soil (Supplementary Figure S6). Previous research showed that several *Cyphellophora* species have been associated with potential pathogens (Decock et al., 2003). Consequently, the co-inoculation of *Bradyrhizobium japonicum* 5038 and *Bacillus aryabhattai* MB35-5 could form a protective microdomain in the soybean rhizosphere to defense against soil-borne fungal pathogens, especially Fusarium (wilt disease) (Fu et al., 2017; Mendes et al., 2018).

The VPA (Supplementary Table S7) and Mantel test (Supplementary Tables S2, S3) all demonstrated that nitrogen content was a vital environment factor for altering the microbial community, and by altering HN and AN contents, the co-inoculation of *Bradyrhizobium japonicum* 5038 and *Bacillus aryabhattai* MB35-5 could change the microbial community and further drive the assembly process in the black and cinnamon soil, respectively. This consistency may be attributed to the increase in the abundance of *Azospirillales* with the co-inoculation of *Bradyrhizobium japonicum* 5038 and *Bacillus aryabhattai* MB35-5 (Supplementary Table S6). According to the above analysis, although the change in the microbial diversity is not significant, the co-inoculation of *Rhizobium* and *Bacillus* can substantially influence the interaction and community construction of soil microorganisms to a great extent via changes in the nitrogen content.

## Conclusion

In conclusion, the co-inoculation of *Bradyrhizobium japonicum* 5038 and *Bacillus aryabhattai* MB35-5 could structure the bacterial and fungi communities, and with an increase in the number of PGPR species, the bacterial and fungal diversity and complexity of the networks increased. The co-inoculation of *Bradyrhizobium japonicum* 5038 and *Bacillus aryabhattai* MB35-5 negligibly influenced the assembly process of the soil bacterial community; however, it exerted a significant impact on the assembly process of the fungal community. Moreover, our results suggest that the inoculation of *Bradyrhizobium japonicum* 5038 increases the deterministic process for fungi, whereas the addition of *Bacillus aryabhattai* MB35-5 to *Bradyrhizobium japonicum* 5038 increases the stochastic process for fungi. The co-inoculation of *Bradyrhizobium japonicum* 5038 and *Bacillus aryabhattai* MB35-5 could limit the process of species dispersal diffusion, which might be one reason for controlling the pathogen invasion. In general, these findings have broadened our understanding of the response of the bacterial and fungi communities on the co-inoculation of *Rhizobium* and *Bacillus* in farmland, which provides a certain reference for the development and application of *Rhizobium* compound inoculants.

## Data Availability Statement

The original contributions presented in the study are publicly available. This data can be found here: NCBI, under accession numbers: PRJNA788980, PRJNA788360.

## Author Contributions

YZ, DG, and JL designed the experiment. YZ analyzed all data and wrote the first draft of the manuscript. XL and G-FG contributed new reagents or analytical tools. DG, FM, BL, and PX performed the field experiment. XJ, MM, FC, LL, and JL edited the manuscript and checked the language. All the authors contributed to manuscript revisions.

## Conflict of Interest

The authors declare that the research was conducted in the absence of any commercial or financial relationships that could be construed as a potential conflict of interest.

## Publisher’s Note

All claims expressed in this article are solely those of the authors and do not necessarily represent those of their affiliated organizations, or those of the publisher, the editors and the reviewers. Any product that may be evaluated in this article, or claim that may be made by its manufacturer, is not guaranteed or endorsed by the publisher.

## Footnotes

1
http://rdp.cme.msu.edu/

2
www.i-sanger.com
