# Supplementary material for: Profound Change in Soil Microbial Assembly Process and Co-occurrence Pattern in Co-inoculation of Bradyrhizobium japonicum 5038 and Bacillus aryabhattai MB35-5 on Soybean
Source: Front Microbiol. 2022 Mar 18;13:846359. doi: 10.3389/fmicb.2022.846359 (PMC8972127; doi:10.3389/fmicb.2022.846359)
Supplement: Supplementary file 1 [file Data_Sheet_1.docx]

**Title:Profound change in soil microbial assembly process and co-occurrence pattern in** **co-****inoculation of *Bradyrhizobium japonicum* 5038 and *Bacillus* *aryabhattai* MB35-5 on soybean**

Yubin Zhao^1^, Dawei Guan ^1^, Xv Liu^2^, Guifeng Gao^2^, Fangang Meng^3^, Bingqiang Liu^4^, Pengfei Xing^1^, Xin Jiang^1^, Mingchao Ma ^1^, Fengming Cao^1^, Li Li^1^, Jun Li^1*^

^1^ Institute of Agricultural Resources and Regional Planning, Chinese Academy of Agricultural Sciences, Beijing, China

^2^ State Key Laboratory of Soil and Sustainable Agriculture, Institute of Soil Science, Chinese Academy of Sciences, Nanjing, China

^3^ Soybean Research Institute, Jilin Academy of Agricultural Sciences, Jilin, China

^4^ Institute of Cereal and Oil Crops, Hebei Academy of Agricultural and Forestry Sciences, Hebei, China

*Corresponding Author: [lijun01@caas.cn](mailto:lijun01@caas.cn),

Institute of Agricultural Resources and Regional Planning, Chinese Academy of Agricultural Sciences, Beijing 100081, PR China.

Tel: +8610 82106208.

FAX: +86 1082108702

**Fig. S1.** Effects of *Bradyrhizobium japonicum 5038* and *Bacillus aryabhattai MB35-5* co-inoculation on nodules of soybean in black soil and cinnamon soil. (a) Pictures of nodules with *Bradyrhizobium japonicum 5038* and *Bacillus aryabhattai MB35-5* inoculation. (b) Nodule numbers and Dry weight of soybean plants under co- inoculation of *Bradyrhizobium japonicum 5038* and *Bacillus aryabhattai MB35-5*. B0, B1, and B2 means no-inoculation, inoculation of *Bradyrhizobium japonicum* 5038, and co-inoculation of *Bradyrhizobium japonicum* 5038 and *Bacillus aryabhattai* MB35-5 in the black soil, and C0, C1, and C2 means no-inoculation, inoculation of *Bradyrhizobium japonicum* 5038, and co-inoculation of *Bradyrhizobium japonicum* 5038 and *Bacillus aryabhattai* MB35-5 in the cinnamon soils.


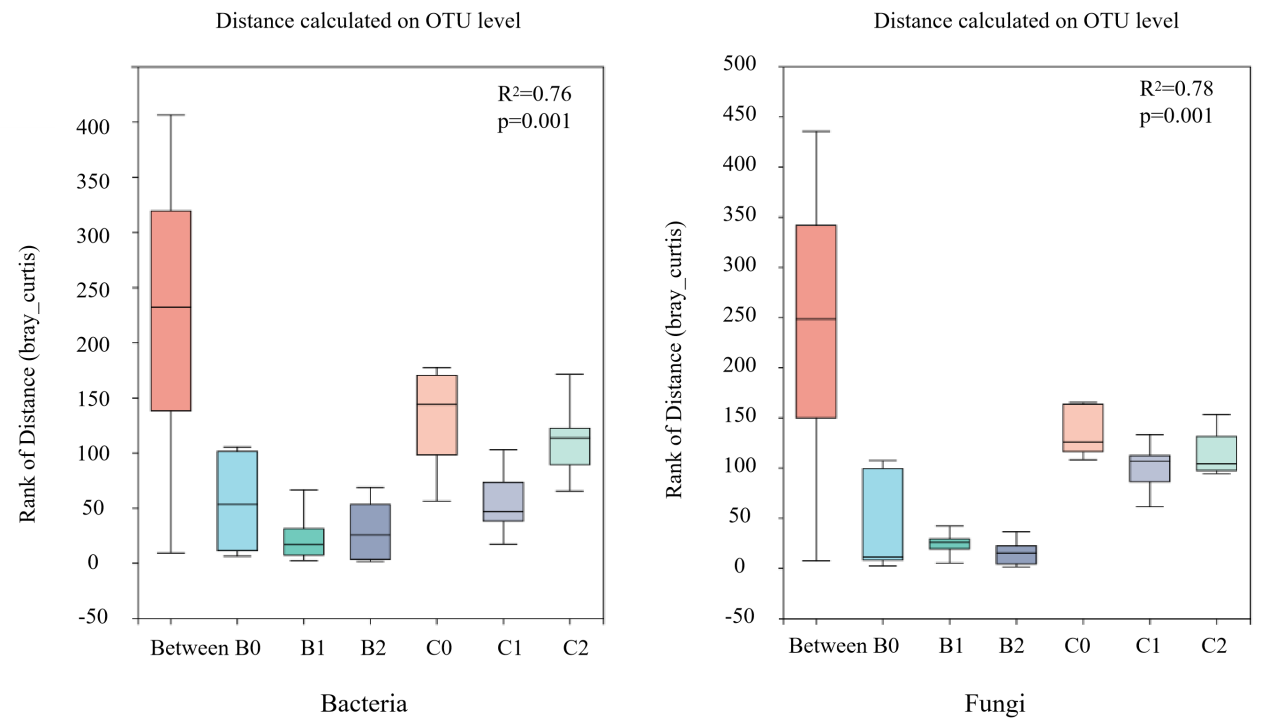


**Fig. S2.** Distance calculated for no-inoculation, one-inoculation and two-inoculation in black (B0, B1, and B2) and cinnamon soils (C0, C1, and C2) of altered bacterial and fungi communities.


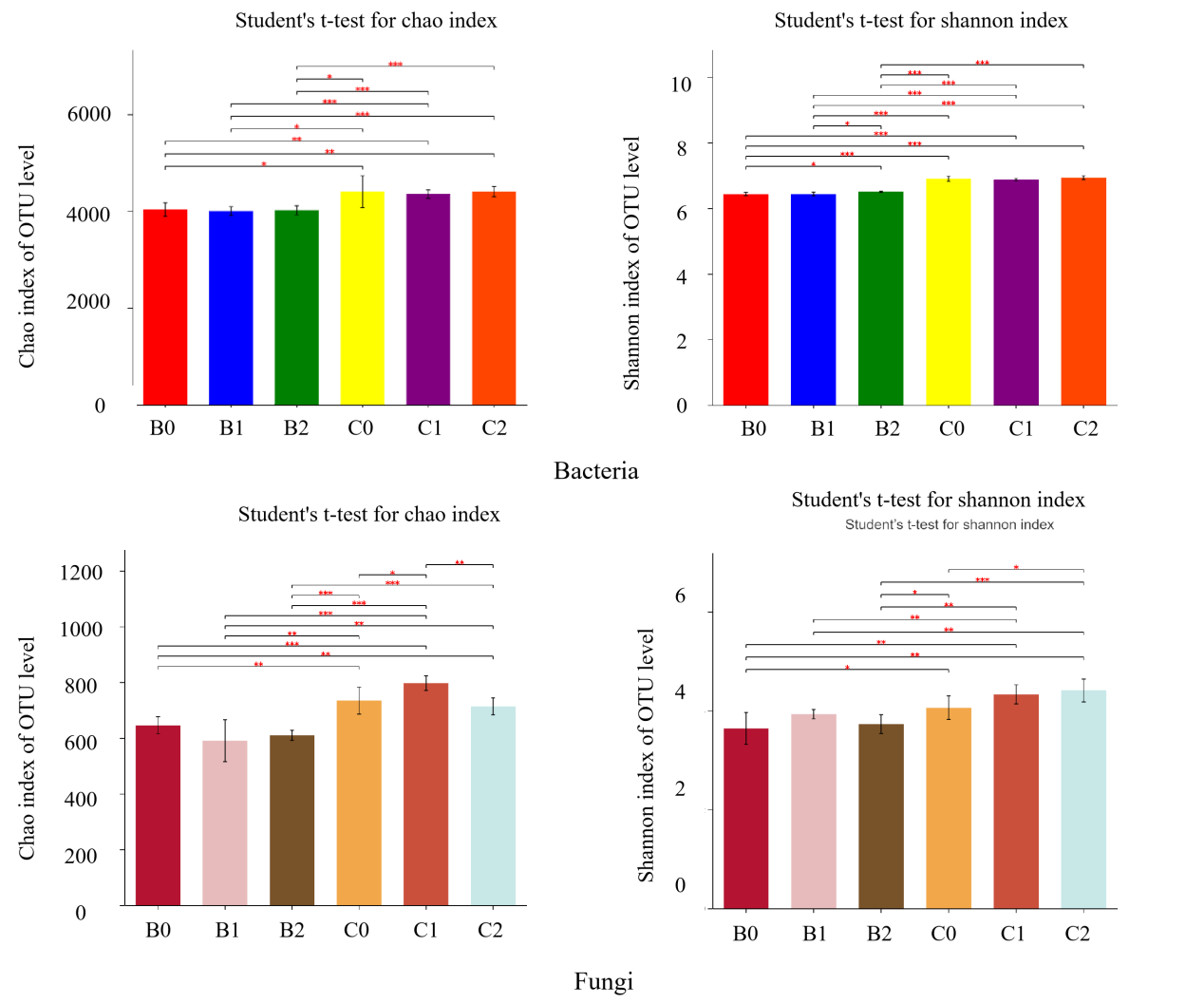


**Fig. S3.** The Chao and Shannon index of α-diversity for no-inoculation, one-inoculation and two-inoculation in black (B0, B1, and B2) and cinnamon soils (C0, C1, and C2). Asterisk in red indicates a significant difference at *p*< 0.05. * *p* < 0.05, ** *p* < 0.01, *** *p*< 0.001.


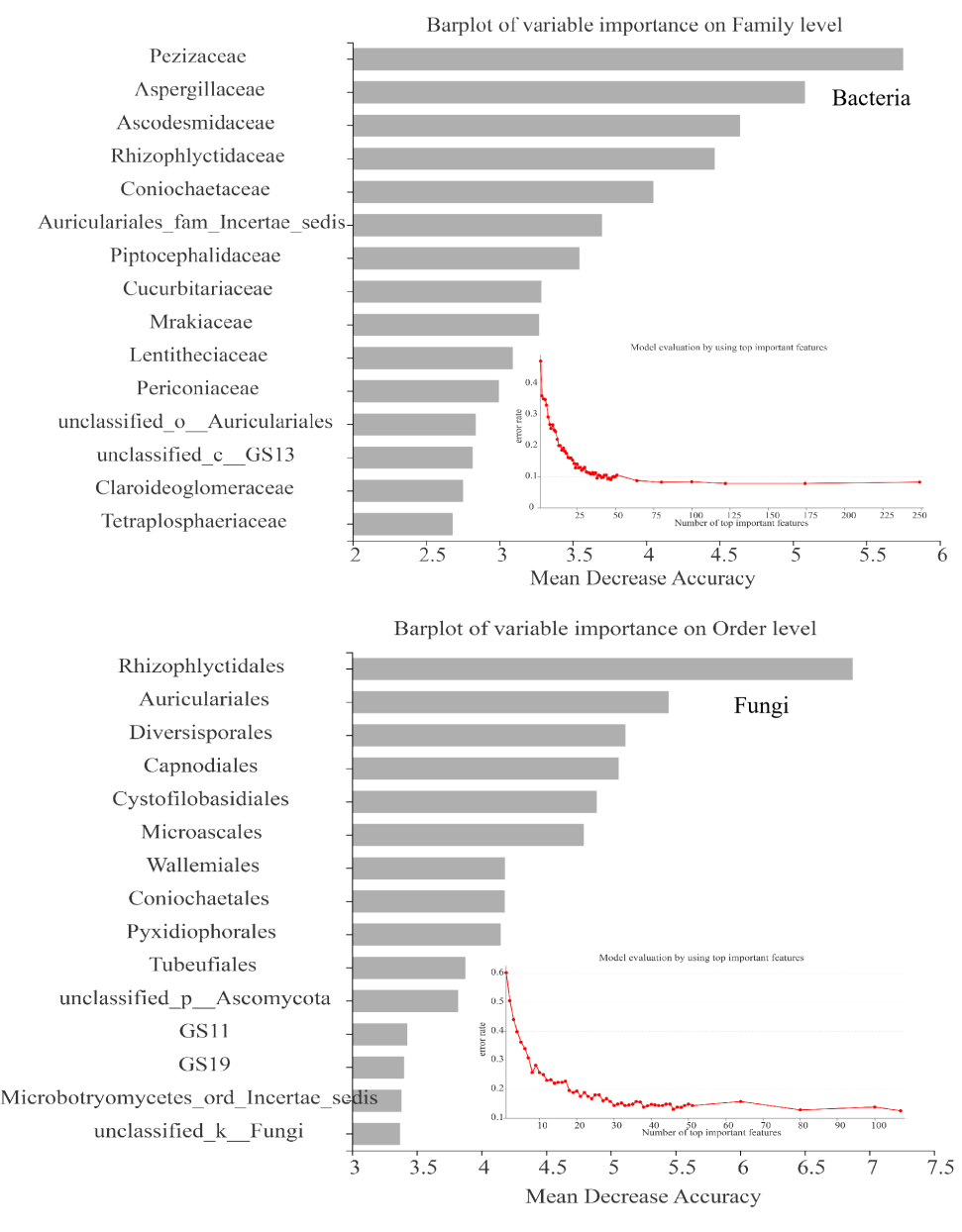


**Fig. S4.** Biomarkers on family and order level for bacteria and fungi. The cross-validation error of 500 times as a function of the number of input orders used to regress order of variable importance. The top 15 biomarkers that identified using random forests regression of their relative abundances are ranked in a descending order of their importance to the accuracy of the model.


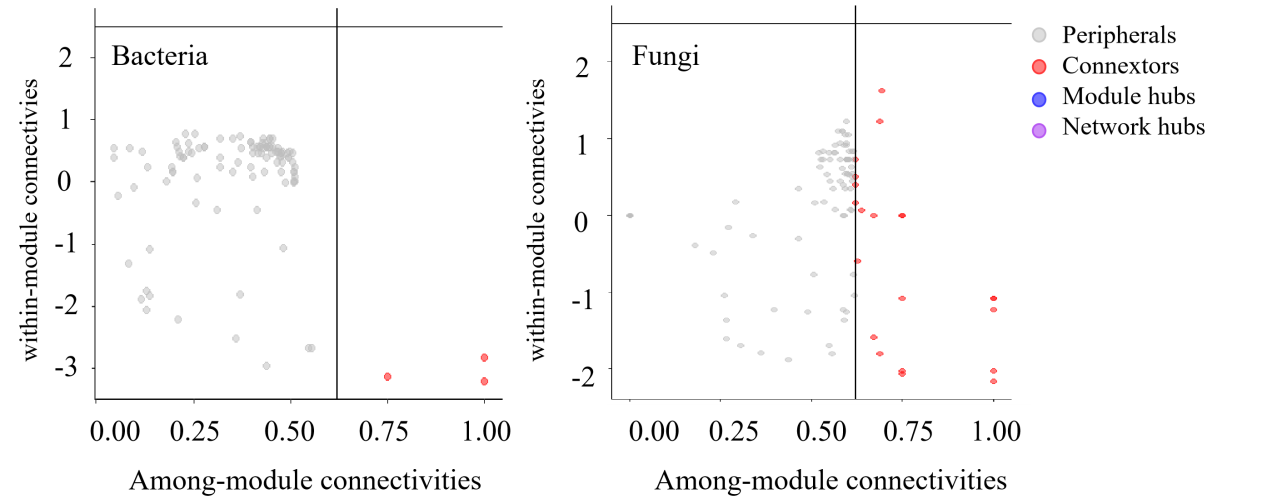


**Fig. S5.** The universal role of each node in co-occurrence network for bacteria and fungi. OTUs after removing the total sum less than 1% in the six samples were analyzed.


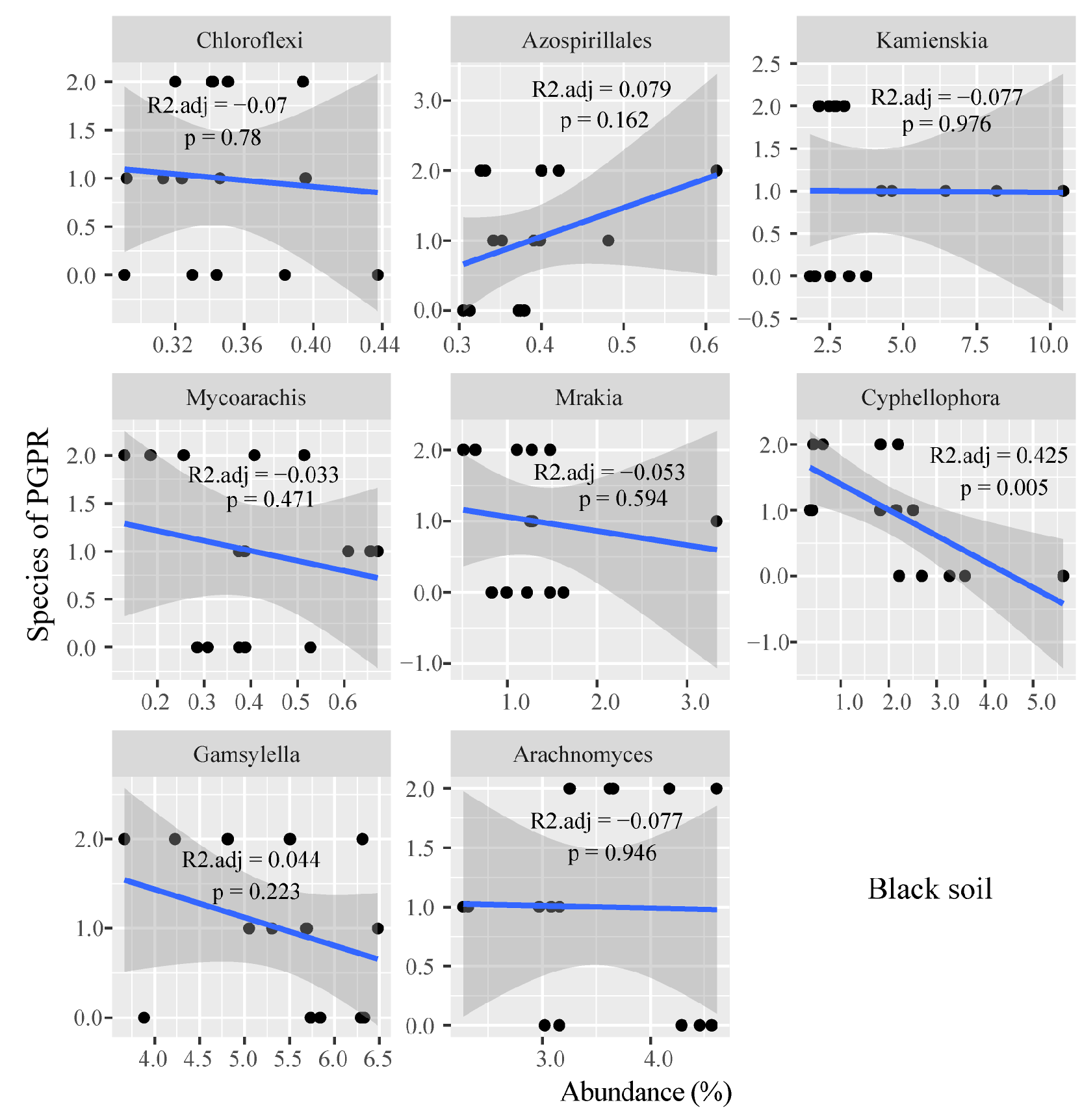


**Fig. S6.** The relationship between species of PGPR and the relative abundance of key microorganisms in black soil. Data were fitted using linear regression and assessed by Spearman's rank correlation ρ.
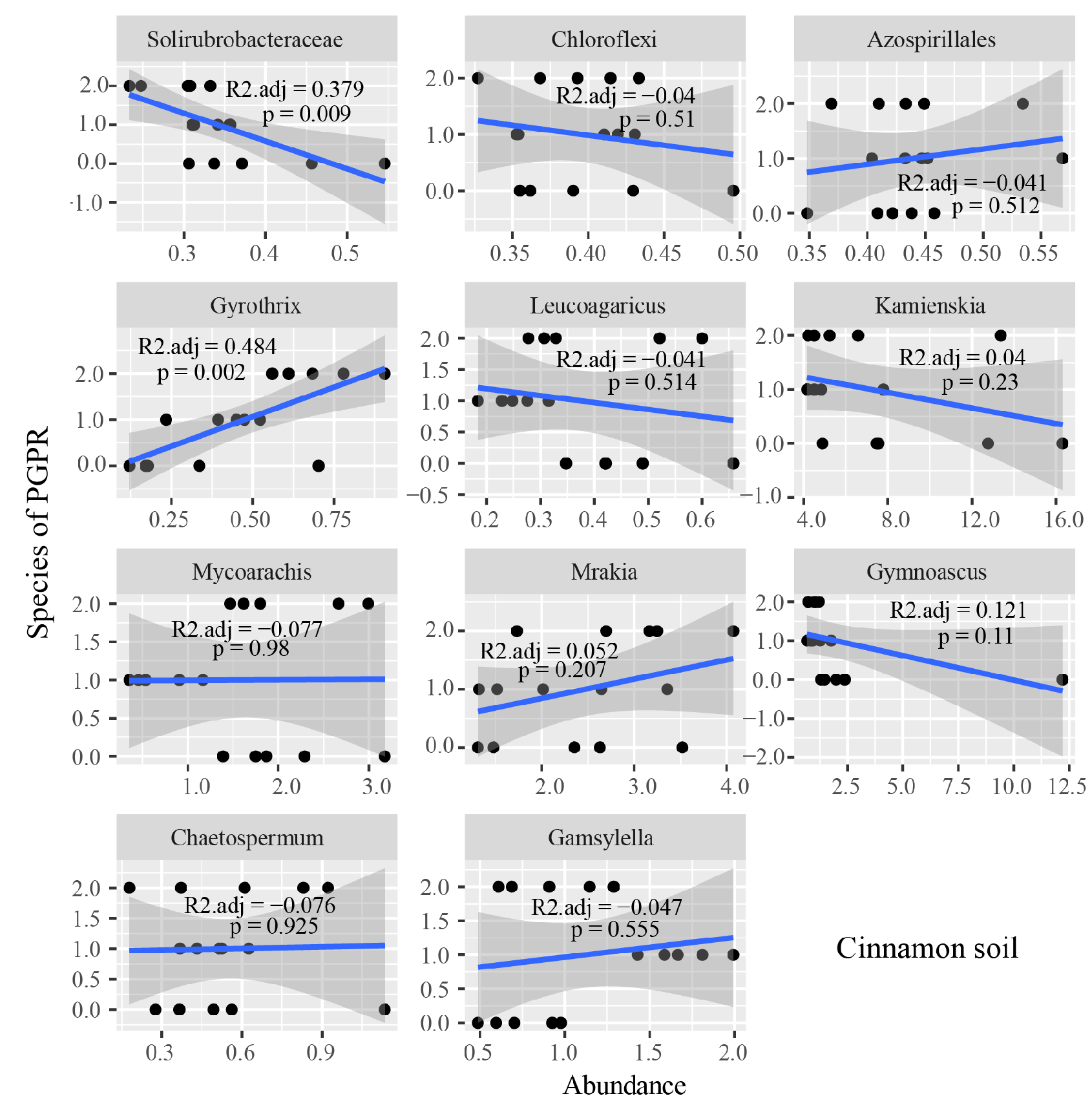


**Fig. S7.** The relationship between species of PGPR and the relative abundance of Keystone species in cinnamon soil. Data were fitted using linear regression and assessed by Spearman's rank correlation


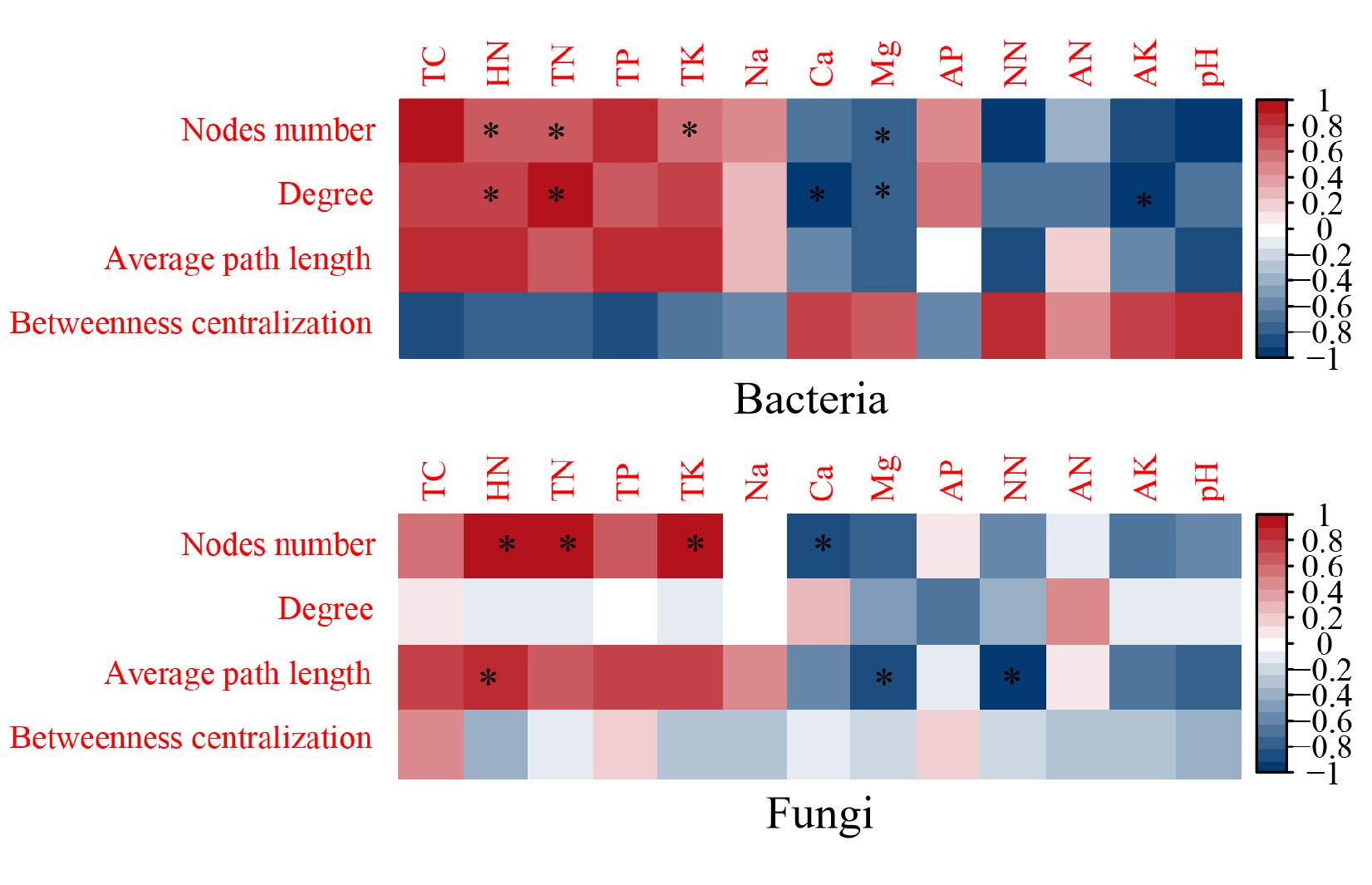


**Fig. S8.** Heatmap showing the relative abundances of the environment factor with
character of co-occurrence network for bacteria and fungi.


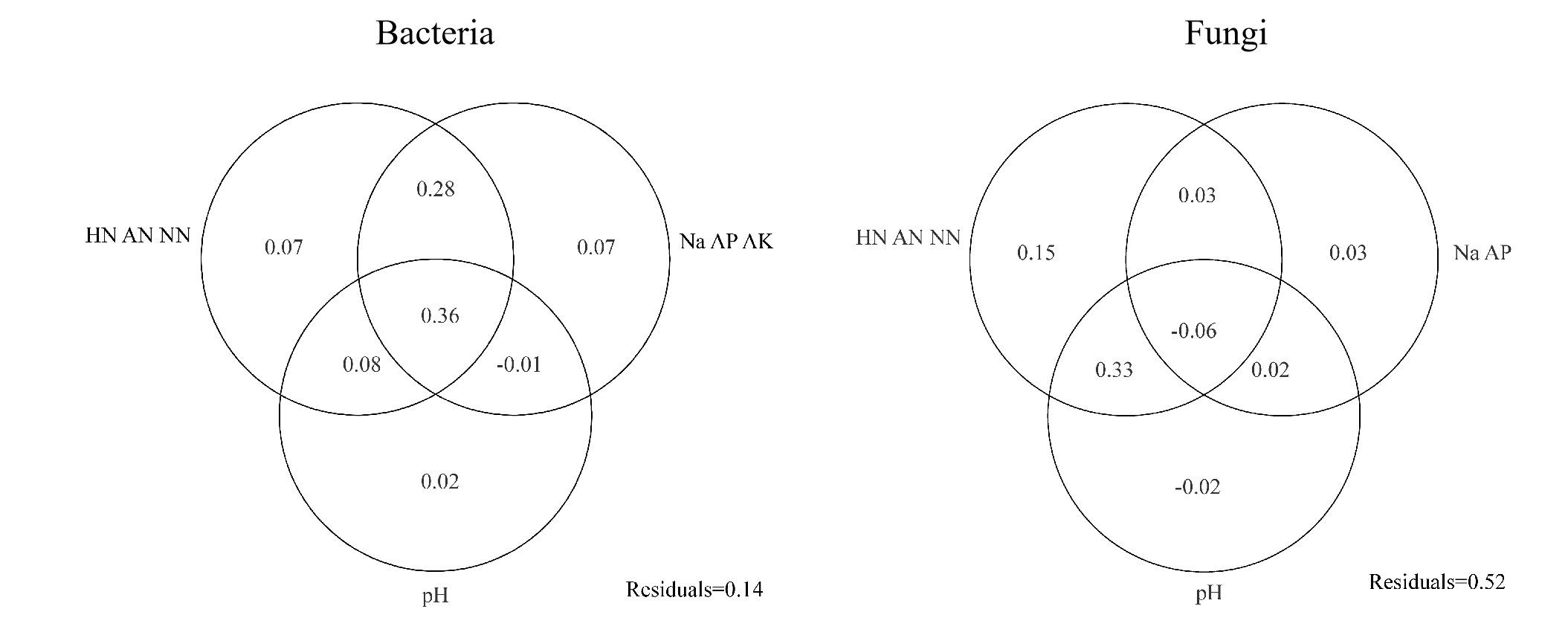


**Fig. S9.** Variation partitioning analysis of bacterial and fungal β-diversity variance explained by environment factor. (Residuals) is the residual variance.

**Table S1.** Soil physicochemical properties. Data are showed as Mean (Std. Error).

| Treatment | B0 | B1 | B2 | C0 | C1 | C2 |
| --- | --- | --- | --- | --- | --- | --- |
| TC (g/kg) | 17.13±0.14 | 17.30±0.23 | 17.10±0.14 | 25.17±0.14 | 23.63±0.23 | 23.37±0.14 |
| TN (%) | 0.11±0.00 | 0.11±0.00 | 0.11±0.00 | 0.14±0.00 | 0.15±0.00 | 0.16±0.00 |
| TP (%) | 0.06±0.00 | 0.06±0.00 | 0.05±0.00 | 0.10±0.02 | 0.09±0.00 | 0.09±0.00 |
| TK (%) | 1.14±0.01 | 1.15±0.01 | 1.15±0.01 | 1.87±0.01 | 1.73±0.17 | 1.95±0.12 |
| HN (mg/kg) | 95.40±4.40 | 93.80±2.25 | 103.00±4.41 | 138.33±8.12 | 137.33±6.09 | 141.67±10.37 |
| NN (mg/kg) | 9.83±0.15 | 9.89±0.06 | 9.32±0.03 | 5.56±0.37 | 7.35±0.25 | 8.79±0.14 |
| AN (mg/kg) | 0.24±0.03 | 0.28±0.06 | 0.31±0.12 | 1.76±0.69 | 1.62±0.09 | 1.48±0.16 |
| AP (mg/kg) | 34.40±0.31 | 34.10±0.42 | 34.80±0.40 | 22.33±0.58 | 20.37±0.46 | 22.63±0.19 |
| AK (mg/kg) | 232.00±2.88 | 238.00±1.03 | 237.00±1.03 | 190.67±61.46 | 136.00±1.55 | 164.67±6.35 |
| Na (%) | 1.47±0.01 | 1.49±0.01 | 1.48±0.01 | 1.82±0.01 | 1.44±0.01 | 1.71±0.01 |
| Ca (%) | 1.90±0.15 | 2.03±0.04 | 2.24±0.17 | 1.44±0.09 | 1.30±0.07 | 1.16±0.14 |
| Mg (%) | 0.55±0.00 | 0.55±0.00 | 0.55±0.00 | 0.35±0.01 | 0.29±0.06 | 0.35±0.02 |
| pH | 7.84±0.01 | 7.83±0.01 | 7.84±0.12 | 7.76±0.02 | 7.79±0.02 | 7.80±0.01 |

Note: B0, B1, and B2 means no-inoculation, inoculation of *Bradyrhizobium japonicum* 5038, and co-inoculation of *Bradyrhizobium japonicum* 5038 and *Bacillus aryabhattai* MB35-5 in the black soil, and C0, C1, and C2 means no-inoculation, inoculation of *Bradyrhizobium japonicum* 5038, and co-inoculation of *Bradyrhizobium japonicum* 5038 and *Bacillus aryabhattai* MB35-5 in the cinnamon soils.

**Table S2.** Mantel test examine the relationships between soil physicochemical properties and soil bacterial community in black soil and cinnamon soil. Value in bold indicates a significant difference at *p*< 0.05.

| Soil Properties | Black soil | | Cinnamon soil | |
| --- | --- | --- | --- | --- |
|  | Mantel *r* | *p* | Mantel *r* | *p* |
| TC ((g/kg)) | **0.24** | **0.02** | **0.53** | **0.00** |
| TN (%) | 0.12 | 0.52 | **0.30** | **0.01** |
| HN (mg/kg) | **0.36** | **0.03** | 0.20 | 0.24 |
| NN (mg/kg) | 0.18 | 0.08 | **0.58** | **0.00** |
| AN (mg/kg) | 0.16 | 0.15 | **0.42** | **0.04** |
| TP (mg/kg) | 0.04 | 0.71 | 0.36 | 0.08 |
| AP (mg/kg) | 0.17 | 0.10 | 0.00 | 0.99 |
| TK (mg/kg) | 0.09 | 0.59 | -0.17 | 0.34 |
| AK (mg/kg) | 0.15 | 0.25 | 0.15 | 0.33 |
| Na (mg/kg) | 0.18 | 0.09 | 0.09 | 0.55 |
| Ca (mg/kg) | **0.28** | **0.01** | **0.31** | **0.05** |
| Mg (mg/kg) | 0.13 | 0.12 | -0.19 | 0.24 |
| pH | 0.21 | 0.20 | **0.34** | **0.01** |

**Table S3.** Mantel test examine the relationships between soil physicochemical properties and soil fungal community in black soil and cinnamon soil. Value in bold indicates a significant difference at *p*< 0.05.

| Soil Properties | Black soil | | Cinnamon soil | |
| --- | --- | --- | --- | --- |
|  | Mantel r | *p* | Mantel r | *p* |
| TC ((g/kg)) | **0.28** | **0.01** | **0.61** | **0.00** |
| TN (%) | 0.38 | 0.08 | **0.40** | **0.00** |
| HN (mg/kg) | -0.17 | 0.32 | -0.14 | 0.36 |
| NN (mg/kg) | **0.48** | **0.00** | **0.68** | **0.00** |
| AN (mg/kg) | **0.30** | **0.01** | 0.23 | 0.17 |
| TP (mg/kg) | -0.02 | 0.87 | 0.28 | 0.09 |
| AP (mg/kg) | **0.33** | **0.00** | **0.23** | **0.04** |
| TK (mg/kg) | **0.22** | **0.04** | -0.12 | 0.43 |
| AK (mg/kg) | 0.22 | 0.06 | **0.34** | **0.01** |
| Na (mg/kg) | **0.41** | **0.00** | **0.32** | **0.01** |
| Ca (mg/kg) | **0.31** | **0.00** | **0.28** | **0.03** |
| Mg (mg/kg) | **0.38** | **0.00** | -0.18 | 0.27 |
| pH | 0.14 | 0.39 | **0.40** | **0.00** |

**Table S4.** The spearman correlation coefficient (ρ) between the relative abundance of top ten phyla or class for bacteria and fungi and the main soil physicochemical properties in black soil. Value in bold indicates a significant difference at *p*< 0.05. * *p* < 0.05, ** *p* < 0.01, *** *p*< 0.001.

|  |  | NN | TP | pH | AK | TN | HN |
| --- | --- | --- | --- | --- | --- | --- | --- |
| Bacteria  (Phylum) | Actinobacteriota | -0.22 | 0.29 | **-0.62 *** | **-0.56*** | -0.01 | -0.38 |
|  | Proteobacteria | -0.04 | -0.16 | -0.18 | **0.54*** | 0.36 | 0.45 |
|  | Chloroflexi | 0.01 | 0.48 | -0.18 | **-0.76 **** | -0.47 | -0.50 |
|  | Acidobacteriota | 0.28 | -0.17 | 0.45 | 0.16 | -0.28 | 0.02 |
|  | Gemmatimonadota | -0.45 | -0.13 | -0.18 | -0.01 | 0.47 | 0.14 |
|  | Firmicutes | 0.15 | -0.04 | -0.38 | 0.48 | 0.15 | 0.24 |
|  | Bacteroidota | -0.04 | -0.18 | -0.18 | 0.43 | 0.39 | 0.42 |
|  | Myxococcota | -0.47 | -0.06 | -0.13 | 0.08 | **0.53*** | 0.16 |
|  | Patescibacteria | -0.03 | **-0.52*** | **0.58 *** | **0.83***** | 0.28 | **0.59*** |
|  | Methylomirabilota | -0.19 | -0.04 | 0.03 | 0.16 | 0.26 | 0.23 |
| Fungi  (Class) | Sordariomycetes | -0.50 | -0.14 | 0.44 | 0.33 | **0.83 ***** | 0.23 |
|  | Dothideomycetes | 0.11 | 0.03 | -0.48 | -0.40 | -0.38 | -0.30 |
|  | Agaricomycetes | 0.49 | 0.21 | -0.06 | -0.06 | **-0.53*** | -0.09 |
|  | Mortierellomycetes | -0.06 | -0.31 | 0.32 | **0.55*** | 0.28 | **0.66**** |
|  | Tremellomycetes | **0.59 *** | 0.40 | **-0.53 *** | -0.08 | **-0.62*** | -0.17 |
|  | Pezizomycetes | 0.02 | 0.36 | -0.39 | **-0.61*** | **-0.52*** | **-0.55*** |
|  | Eurotiomycetes | 0.45 | 0.20 | -0.46 | 0.09 | -0.46 | -0.22 |
|  | Leotiomycetes | -0.07 | -0.34 | 0.24 | 0.22 | 0.24 | **0.54*** |

**Table S5.** The spearman correlation coefficient (ρ) between the relative abundance of top ten phyla or class for bacteria and fungi and the main soil physicochemical properties in cinnamon soil. Value in bold indicates a significant difference at *p*< 0.05. * *p* < 0.05, ** *p* < 0.01, *** *p*< 0.001.

|  |  | HN | TN | Na | Ca | Mg | AN | pH |
| --- | --- | --- | --- | --- | --- | --- | --- | --- |
| Bacteria  (Phylum) | Acidobacteriota | 0.07 | 0.02 | -0.22 | -0.30 | **-0.59 *** | 0.03 | 0.21 |
|  | Proteobacteria | -0.20 | -0.15 | -0.08 | 0.19 | 0.17 | 0.00 | -0.35 |
|  | Actinobacteriota | -0.30 | **-0.39 *** | 0.46 | 0.60 | **0.59*** | 0.13 | -0.33 |
|  | Chloroflexi | **-0.01*** | **-0.55*** | 0.59 | 0.58 | 0.28 | **0.33**** | -0.52 |
|  | Bacteroidota | 0.29 | 0.55 | 0.04 | -0.37 | 0.16 | -0.50 | 0.26 |
|  | Myxococcota | -0.03 | 0.44 | -0.03 | -0.25 | 0.35 | -0.37 | 0.18 |
|  | Gemmatimonadota | 0.19 | 0.23 | **0.46*** | -0.22 | 0.25 | -0.27 | 0.04 |
|  | Patescibacteria | -0.06 | 0.03 | -0.43 | -0.13 | **-0.53*** | 0.04 | **-0.06*** |
|  | Methylomirabilota | -0.30 | -0.13 | 0.06 | 0.22 | -0.10 | -0.12 | -0.03 |
|  | Firmicutes | 0.18 | **0.54*** | 0.01 | -0.52 | 0.21 | **-0.33*** | 0.45 |
| Fungi  (Class) | Sordariomycetes | 0.17 | -0.13 | 0.26 | 0.40 | 0.49 | -0.07 | **-0.54*** |
|  | Dothideomycetes | 0.01 | 0.34 | -0.41 | -0.15 | 0.03 | -0.20 | 0.26 |
|  | Agaricomycetes | 0.22 | 0.48 | **-0.56 *** | -0.51 | -0.33 | -0.20 | **0.59*** |
|  | Mortierellomycetes | -0.26 | 0.08 | **-0.53*** | -0.09 | **-0.64*** | -0.12 | 0.14 |
|  | Eurotiomycetes | -0.01 | 0.47 | -0.44 | -0.44 | -0.08 | -0.06 | **0.56 *** |
|  | Tremellomycetes | -0.21 | **0.53*** | **-0.78***** | **-0.53*** | **-0.62 *** | -0.21 | **0.57*** |
|  | Pezizomycetes | 0.10 | **0.72**** | -0.18 | **-0.57*** | 0.17 | -0.40 | **0.67**** |

**Table S6.** The nodes that identified as connector hub and connector in the co-occurrence network for bacteria and fungi. The remaining 13 fungi and 3 bacteria were selected as biomaker which the total abundance was more than 5%.

|  | Order/Phylum | Genus | B0 (%) | B1 (%) | B2 (%) | C0 (%) | C1 (%) | C2 (%) |
| --- | --- | --- | --- | --- | --- | --- | --- | --- |
| Fungi | Glomerellales | *Gyrothrix* | 0.36 | 0.16 | 0.17 | 0.30 | 0.42 | 0.71 |
|  | Glomerellales | *Gymnoascus* | 0.08 | 0.10 | 0.09 | 3.87 | 1.10 | 0.96 |
|  | Helotiales | *Cyphellophora* | 3.47 | 1.45 | 1.10 | 0.04 | 0.13 | 0.83 |
|  | Hypocreales | *Kockovaella* | 0.39 | 0.31 | 0.09 | 0.05 | 0.19 | 0.12 |
|  | Hypocreales | *Leucoagaricus* | 0.25 | 0.30 | 0.29 | 0.47 | 0.25 | 0.41 |
|  | Hypocreales | *Leptosphaeria* | 0.27 | 0.29 | 0.33 | 0.07 | 0.08 | 0.34 |
|  | Hypocreales | *Kondoa* | 0.16 | 0.21 | 0.14 | 0.41 | 0.31 | 0.25 |
|  | Hypocreales | *Kamienskia* | 2.65 | 6.79 | 2.60 | 9.80 | 5.10 | 6.78 |
|  | Pezizales | *Gamsylella* | 5.62 | 5.64 | 4.90 | 0.74 | 1.70 | 0.93 |
|  | Pleosporales | *Arachnomyces* | 3.90 | 2.76 | 3.86 | 0.01 | 0.04 | 0.04 |
|  | Sordariales | *Mycoarachis* | 0.38 | 0.54 | 0.30 | 2.10 | 0.68 | 2.11 |
|  | Sordariales | *Mrakia* | 1.23 | 1.68 | 1.00 | 2.26 | 2.17 | 2.98 |
|  | Tubeufiales | *Chaetospermum* | 0.08 | 0.06 | 0.21 | 0.57 | 0.49 | 0.58 |
| Bacteria | Actinobacteriota | *Solirubrobacteraceae* | 0.15 | 0.35 | 0.29 | 0.40 | 0.34 | 0.29 |
|  | Chloroflexi | *Chloroflexi* | 0.36 | 0.33 | 0.35 | 0.41 | 0.39 | 0.39 |
|  | Proteobacteria | *Azospirillales* | 0.35 | 0.39 | 0.42 | 0.41 | 0.46 | 0.44 |

**Table S7** Variation partitioning analysis (VPA) of bacterial and fungal β-diversity variance explained by environment factor.

|  | Environment factor | R² |
| --- | --- | --- |
| Bacteria | HN | 75.90% |
|  | AN | 1.66% |
|  | pH | 44.21% |
|  | TN | 76.79% |
|  | Na | 3.24% |
|  | Ca | 55.16% |
|  | Mg | 77.28% |
| Fungi | HN | 40.52% |
|  | AN | 0.53% |
|  | pH | 26.67% |
|  | Na | 1.43% |
|  | AP | 0.69% |
|  | NN | 26.58% |
